# Multiple New Paralytic Shellfish Toxin Vectors in Offshore North Sea Benthos, a Deep Secret Exposed

**DOI:** 10.3390/md18080400

**Published:** 2020-07-29

**Authors:** Karl J. Dean, Robert G. Hatfield, Vanessa Lee, Ryan P. Alexander, Adam M. Lewis, Benjamin H. Maskrey, Mickael Teixeira Alves, Benjamin Hatton, Lewis N. Coates, Elisa Capuzzo, Jim R. Ellis, Andrew D. Turner

**Affiliations:** 1 Centre for Environment Fisheries and Aquaculture Science (Cefas), Barrack Road, Weymouth, Dorset DT4 8UB, UK; Robert.Hatfield@Cefas.co.uk (R.G.H.); Vanessa.Lee@cefas.co.uk (V.L.); Ryan.Alexander@cefas.co.uk (R.P.A.); Adam.Lewis@cefas.co.uk (A.M.L.); Ben.Maskrey@cefas.co.uk (B.H.M.); Mickael.teixeiraalves@cefas.co.uk (M.T.A.); Lewis.Coates@cefas.co.uk (L.N.C.); elisa.capuzzo@cefas.co.uk (E.C.); andrew.turner@cefas.co.uk (A.D.T.); 2 Department of Chemistry, University of Surrey, Guildford, Surrey GU2 7XH, UK; 3 Centre for Environment Fisheries and Aquaculture Science (Cefas), Pakefield Road, Lowestoft, Suffolk NR33 0HT, UK; benjamin.hatton@cefas.co.uk (B.H.); Jim.Ellis@cefas.co.uk (J.R.E.)

**Keywords:** paralytic shellfish toxins, benthic organisms, starfish, saxitoxins, sunstar, sea chervil

## Abstract

In early 2018, a large easterly storm hit the East Anglian coast of the UK, colloquially known as the ‘Beast from the East’, which also resulted in mass strandings of benthic organisms. There were subsequent instances of dogs consuming such organisms, leading to illness and, in some cases, fatalities. Epidemiological investigations identified paralytic shellfish toxins (PSTs) as the cause, with toxins present in a range of species and concentrations exceeding 14,000 µg STX eq./kg in the sunstar *Crossaster papposus*. This study sought to better elucidate the geographic spread of any toxicity and identify any key organisms of concern. During the summers of 2018 and 2019, various species of benthic invertebrates were collected from demersal trawl surveys conducted across a variety of locations in the North Sea. An analysis of the benthic epifauna using two independent PST testing methods identified a ‘hot spot’ of toxic organisms in the Southern Bight, with a mean toxicity of 449 µg STX eq./kg. PSTs were quantified in sea chervil (*Alcyonidium diaphanum*), the first known detection in the phylum bryozoan, as well as eleven other new vectors (>50 µg STX eq./kg), namely the opisthobranch *Scaphander lignarius*, the starfish *Anseropoda placenta, Asterias rubens, Luidia ciliaris*, *Astropecten irregularis* and *Stichastrella rosea*, the brittlestar *Ophiura ophiura*, the crustaceans *Atelecyclus rotundatus* and *Munida rugosa*, the sea mouse *Aphrodita aculeata*, and the sea urchin *Psammechinus miliaris*. The two species that showed consistently high PST concentrations were *C. papposus* and *A. diaphanum*. Two toxic profiles were identified, with one dominated by dcSTX (decarbamoylsaxitoxin) associated with the majority of samples across the whole sampling region. The second profile occurred only in North-Eastern England and consisted of mostly STX (Saxitoxin) and GTX2 (gonyautoxin 2). Consequently, this study highlights widespread and variable levels of PSTs in the marine benthos, together with the first evidence for toxicity in a large number of new species. These findings highlight impacts to ‘One Health’, with the unexpected sources of toxins potentially creating risks to animal, human and environmental health, with further work required to assess the severity and geographical/temporal extent of these impacts.

## 1. Introduction

**Figure 1:**
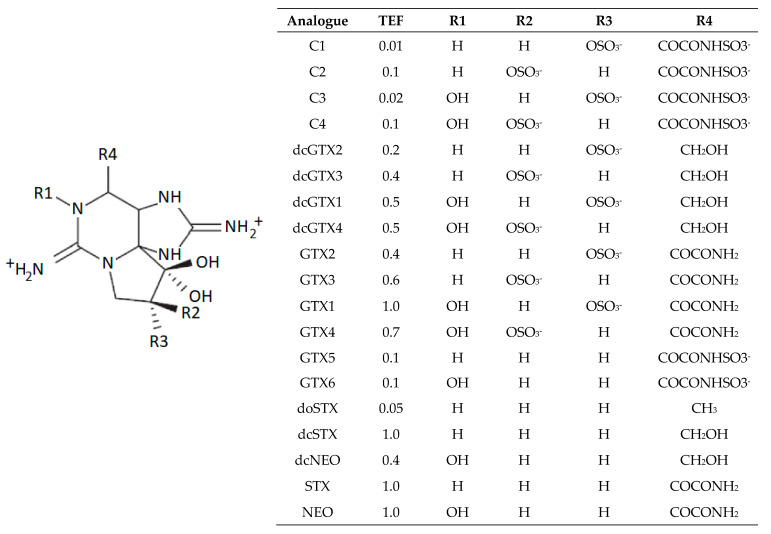
Chemical structures of the commonly-reported Paralytic Shellfish Toxins (TEF = Toxicity Equivalence Factor [10]).

Paralytic Shellfish Poisoning (PSP) is the human illness commonly associated with the consumption of seafood that have bioaccumulated Paralytic Shellfish Toxins (PST) primarily in, but not limited to, bivalve molluscs [1,2]. Production of these toxins are associated with the formation of Harmful Algal Blooms (HABs), which are caused by certain specific phytoplankton species in marine environments [3]. Specific species of cyanobacteria have also been implicated in the production of PSTs from freshwater environments [4]. The parent compound saxitoxin (STX), together with many structurally-related analogues [5,6] (Figure 1), are powerful neurotoxins that bind to site 1 of the Na^+^ voltage gated channel [5], blocking synaptic transmission. Symptoms in humans include vomiting, headaches, dizziness, numbness and tingling of extremities, ataxia and paralysis, and in severe intoxications can cause paralysis and death [7]. Consequently, to limit the risk of human consumption of contaminated bivalve molluscs, regulatory testing for the presence of PSTs is a requirement in a number of nations [8], with a maximum permitted level (MPL) of 800 µg STX eq/ kg of flesh, defined in legislation [9], with any bivalve molluscs exhibiting total PST toxicity above this threshold banned from commercial harvest and human consumption.

There are a number of cyanobacterial genera known to produce saxitoxins [11,12,13,14] of these some proliferate in the planktonic phase and others benthic. Benthic cyanobacterial blooms are typically associated with freshwater and marginal marine habitiats [11,15,16,17]. Benthic genera such as Lyngbya have both saxitoxin producing species [11,18] and marine examples [15], however, marine species are not currently known to produce saxitoxins and to the authors knowledge, none of the known PST producing cyanobacteria are present in freshwater bodies in the UK. Of the dinoflagellate PST producers known globally, the genus *Alexandrium* is well represented in UK waters [19,20], with the other PST producing species, *Pyrodinium bahamense* var. *compressum* and *Gymnodinium catenatum* being absent. Of the species of *Alexandrium* present in UK waters, three have been associated with PST production, *A. minutum, A. catenella* and *A. ostenfeldii* [21]. Within the UK, both *A. catenella* [19] and *A. minutum* [22] have been demonstrated to produce PST at high enough concentrations to cause human health issues in shellfish, whilst local populations of *A. ostenfeldii* have been found to produce only trace levels of PST [19]. The genus *Alexandrium* is also a well-documented producer of resilient cysts [21]. Cyst deposits can be dense [23], and span wide geographic areas [23,24,25]. Grazing on cysts has been shown to be a route of toxin accumulation in shellfish [26]. This suggests that benthic organisms, especially those feeding in sediment or filter feeding from the water directly over the sediment, could be exposed to PST if the cysts are ingested during feeding. Consequently, if filter feeders became toxic, in this way, it would create a pathway for PSTs to accumulate in other benthic organisms and benthic feeders, which prey upon these filter feeders.

While, the risk to humans from PSTs is wellunderstood [27], and management systems are in place to limit the risk of intoxications from shellfish consumption, PSTs can also impact animal health with negative effects on a wide range of marine organisms [28], including fish [29,30,31], whales and seals [32,33], otters [34], sea birds [35,36], sea urchins [37], starfish [38], gastropods [39], as well as bivalve molluscs [40,41]. Furthermore, there have been instances of PSTs affecting terrestrial animals. Notably, after a large winter storm in January 2018 whereby multiple intoxications including two fatalities were reported in dogs walking along East Anglian (UK) beaches, which ingested washed up benthic species that were subsequently found to contain high concentrations of PSTs [42]. Various benthic species were discovered to have accumulated toxins including crabs, flatfish (*Limanda limanda*) and starfish. The total PST concentration in one sunstar (*Crossaster popposus*) sample exceeded 14,000 µg STX eq./kg, representing a concentration 18 times higher than the MPL [9]. The presence of PSTs in this environment at this time was unexpected, as toxin outbreaks in classified shellfish harvesting areas typically happen in the south west of England and the west coast of Scotland from March to September [43]. Quantifiable concentrations of PSTs have never been reported in bivalves molluscs from the south east of England, and between 2008 to 2013, no PSTs in shellfish had been detected anywhere across the UK in January [43]. In addition to the unusual spatial and temporal prevalence, the toxin profiles quantified in the stranded organisms were novel. The typical *A. catenella* [19] (formerly *A. tamarense*) profile found in Scotland typically consists of a high proportion of the gonyautoxins (GTXs) 1&4, with lower proportions of neosaxitoxin (NEO), GTX2&3 and saxitoxin (STX) [43]. *A. minutum* [20,22] found in SW England consistently produces a GTX2&3 and STX profile. The profile determined in the samples associated with the dog intoxications, however, was dominated by decarbamoylsaxitosin (dcSTX) with low relative proportions of STX, gonyautoxin 5 (GTX5) and deoxydecarbamoylsaxitoxin (doSTX). Consequently, the profile resembled the decarbaomyl PST dominated profiles that have undergone enzymatic change, commonly seen in some clam species [44,45], suggesting the possible presence of the transformative enzymes carbomylase [46], and/or sulfocarbamolyase [47] or action by certain bacterial species [48]. Furthermore, the invertebrate species that were implicated were also unexpected. Whilst the accumulation of PSTs in marine organisms other than bivalve molluscs is well-documented [2,32,49,50,51,52,53,54], PSTs presence in starfish is rarely described [53,55,56,57,58], with toxicity generally far lower than the 14,000 µg STX eq./kg described in England. The presence of highly toxic starfish in the UK benthos in the winter, in a geographic location where PSTs are rarely, if ever, seen in shellfish, with a toxin profile unlike any currently known domestic algal PST producer, required further investigation to help disseminate which/ if any other organisms are implicated and the geographical range of the toxic benthos.

With a potential novel source of high concentrations of PSTs from the benthos around the East coast of the UK, consideration should also be given to the potential impacts on animal and environmental health, in addition to the potential for human intoxications that may result from the trophic transfer to edible species of these harmful toxins. Consequently, the presence of these toxins in the benthic epifauna in eastern England represents a real threat to ‘One Health’ which needs to be explored.

Overall, there is a clear requirement to understand and document the presence of PSTs in the offshore benthos, to investigate any spatial and temporal differences, determine the species most commonly linked to toxin presence and ultimately determine the level of risk to ‘One Health’ in the UK marine environment.

## 2. Results

### 2.1. PST Toxicity

**Figure 2:**
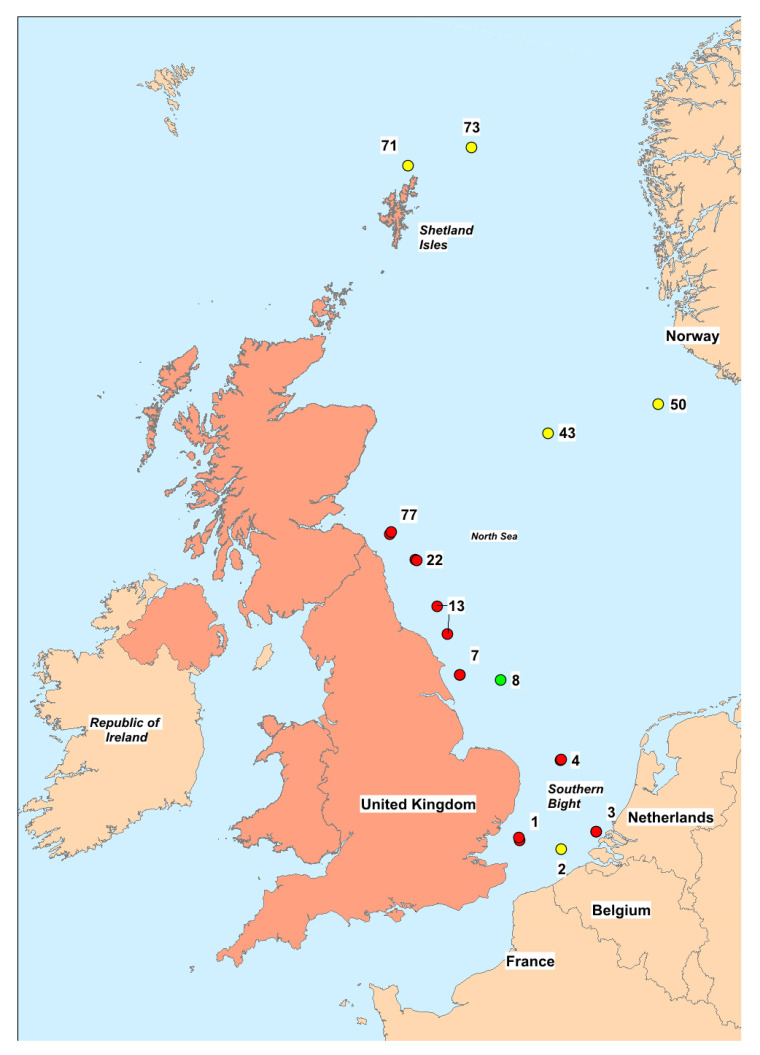
Map indicating locations of each of the sampling locations. Green dots represent sampling locations used in 2018 only, yellow dots represent locations used in 2019 only, and red dots represent locations used in both years. Numbers represent specific the fixed station numbers of the sites. Additional station data can be found in the Appendix A.

PSTs were detected and quantified in samples of benthic marine organisms collected in both 2018 and 2019, although total PST toxicity varied greatly, depending on location, year and species. Table A1 in the Appendix A summarises the total PST toxicity data quantified using two independent detection methods for all samples processed. In total, 168 samples consisting of more than 30 identified species were tested (65 samples from 2018 and 103 samples from 2019), with toxins detected above the limit of detection (LOD) in 61% of all samples (Table 1). The mean toxicity of all samples tested across both years was 127 µg STX eq./kg. Results ranged from non-detects to 2,091 µg STX eq./kg quantified in a bryozoan sampled from Station 3 (Dutch waters) in 2019 (Figure 2). PSTs were also quantified in starfish, sunstars, crabs, sea mouse, gastropods, anemones, urchins, bivalve molluscs and shrimp.

### 2.2. Method Comparison

Wherever possible, both liquid chromatography with tandem mass spectrometry (LC-MS/MS) and liquid chromatography with fluorescence detection (LC-FLD) methods were used to detect and quantify PSTs in each sample, to provide extra confidence in any results produced, given that neither method is formally validated for non-bivalve shellfish species. Quantified concentrations, using both methods, were compared for all samples. No significant difference was determined between total PST concentrations at the 95% confidence level, with a positive correlation for the linear regression (r^2^ = 0.87) evidencing a good agreement between the two methods, with the means tested (calculated as the mean LC-FLD/LC-MS/MS ratio in samples > 80 µg STX eq./kg) difference between the methods showing a 15% positive bias towards the LC-MS/MS method (Table 2). It is also noted that the LC-MS/MS method is also capable of detecting Tetrodotoxin (TTX) and other TTX analogues, as opposed to the LC-FLD. In this study, TTX was not detected in any of the benthic samples from either year.

### 2.3. Toxin Profiles

**Figure 3:**
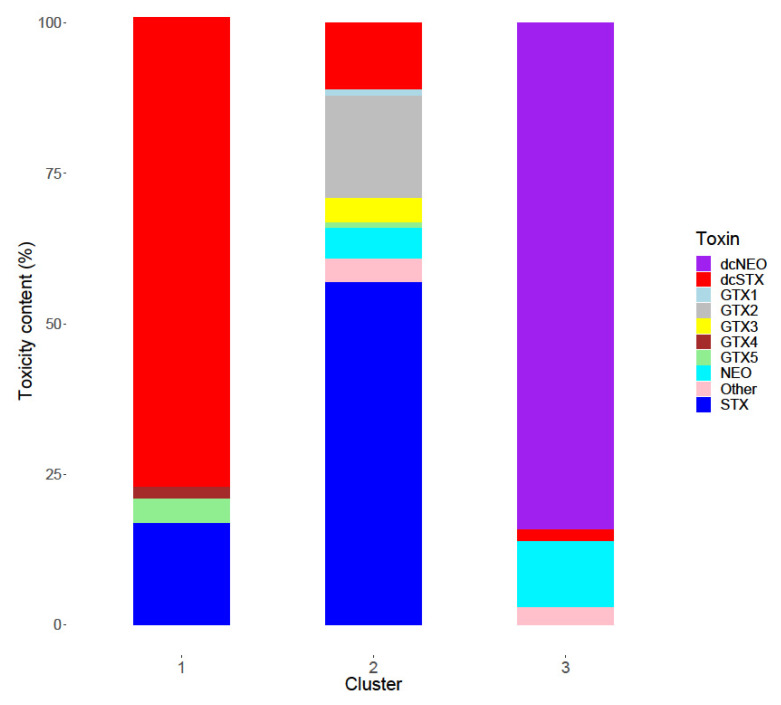
Mean toxin profiles using K-means clustering for all samples with a toxin content >80 µg STX eq./kg. Toxic content described as the percentage of total toxicity in µg STX eq./kg.

K-means clustering as detailed in [61,62] was applied to the LC-MS/MS-derived data for all samples with a total toxicity >80 µg STX eq./kg (n = 35) (Figure 3), with the analysis identifying three distinct toxin profiles based on STX equivalents. Cluster one was associated with a high proportion (78% of total toxin content) of dcSTX with lower relative concentrations of GTX5 (4%), STX (17%) and trace levels of other toxins (1%). This profile was associated with samples obtained from sites in the Southern Bight (Stations 1–3) and sites in the more western parts of the North Sea (Stations 71, 73 and 77) and was the most common profile (49% of all samples > 80 µg STX eq./kg). The second cluster was dominated by STX (57% of toxicity) with smaller proportions associated with GTX2 (17%), dcSTX (11%), NEO (5%), GTX3 (4%) and other toxins (6%). Only two sampling locations (7 and 13) exhibited this profile, which was associated with 42% of the samples >80 µg STX eq./kg. All positive samples from both these sites and both years showed this profile. The remaining 8% of samples (n = 3) centred around the third cluster which contained dcNEO (84%), with a smaller NEO (11%) constituent. These samples were all sourced from Station 1 in 2018.

### 2.4. Inter-Group Variability

**Figure 4:**
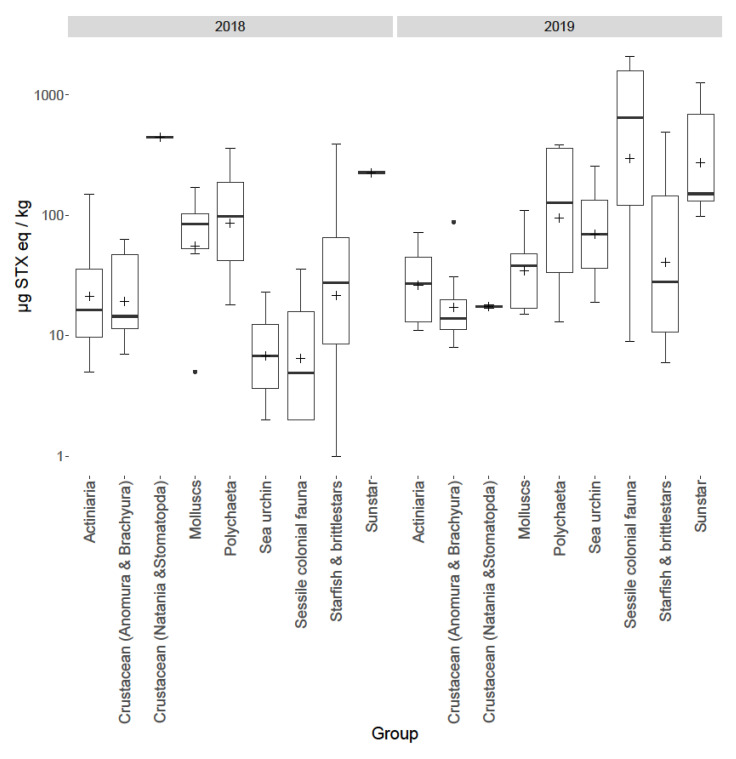
Box and whisker plot highlighting group means (cross), 1st and 3rd quartiles, outliers (dots). and inter quartile ranges for all groups sampled from 2018 and 2019.

Due to the random nature of the species sampling, over 30 different identified species were collected and analysed, with some species collected only once. Consequently, the samples consisting of different species were catalogued into similar taxonomic groups and assessed together to make broad comparisons (Table 1 and Figure 4). The assessment of total toxin concentrations for each group highlighted large variability, with sunstars (*C. papposus*) and sessile fauna showing the highest toxicities. All sunstars analysed across both years contained PSTs regardless of location, from the East coast of England (Station 7) to the Scottish coast (Station 73). Toxicities ranged from 98 to 1275 µg STX eq./kg with a mean of 448 µg STX eq./kg (n = 7)). One sessile fauna sample from Station 3 in 2019 was found to contain the highest toxicity (2090 µg STX eq./kg) out of all samples analysed. Trace toxin concentrations were found in all other taxonomic groups, with the lowest mean toxicity determined in crustaceans (Anomura and Brachyura) (25 µg STX eq./kg) and the lowest occurrence in sea urchins (36%).

### 2.5. Spatial and Temporal Variability

**Figure 5:**
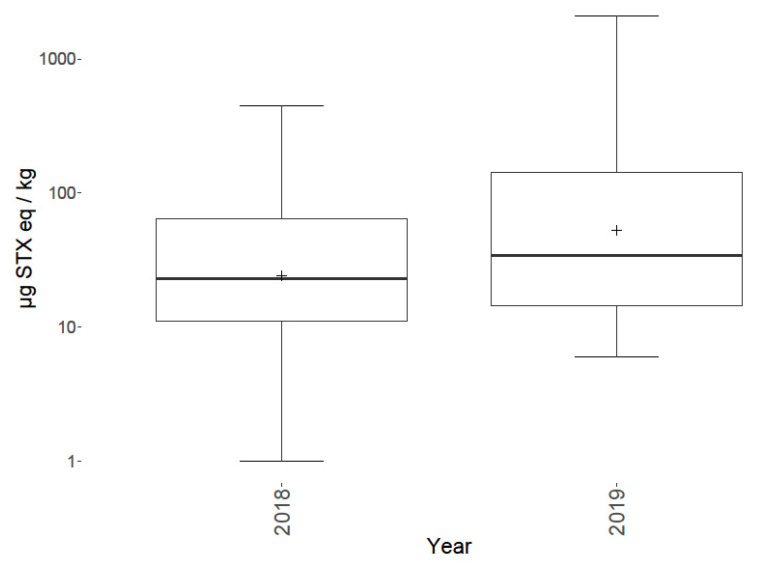
Box and whisker plot highlighting group means (cross), 1st and 3rd quartiles, outliers (dots) and inter quartile ranges for total toxicity for all samples, separated into each year.

**Figure 6:**
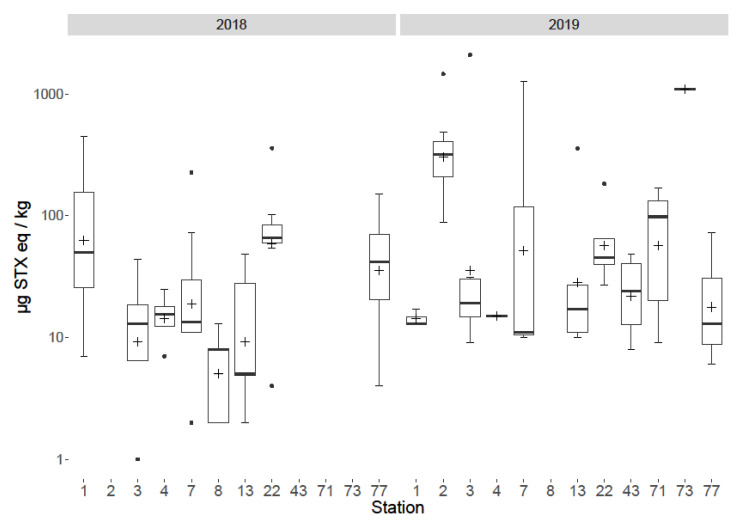
Box and whisker plot highlighting station means (cross), 1st and 3rd quartiles, outliers (dots) and inter quartile ranges for all samples at all stations for 2018 and 2019 separately.

**Figure 7:**
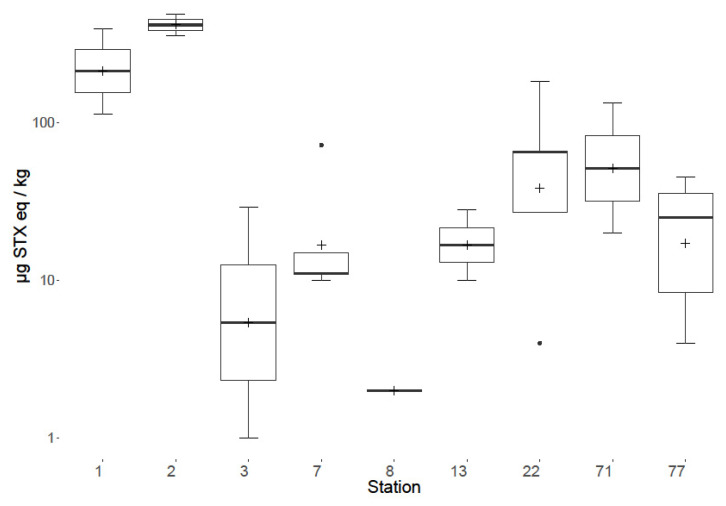
Box and whisker plot highlighting group means (cross), 1st and 3rd quartiles, outliers (dots) and inter quartile ranges of all samples within the Starfish and brittlestars group (excluding sunstars) from all stations for 2018 and 2019 combined.

Toxins were detected in benthic fauna in samples taken during both years at multiple locations. Temporal variability was observed between 2018 and 2019 both overall (Figure 5), on a species level (Figure 4) and spatially (Figure 6). Overall, the total PST levels appeared to increase from 2018 to 2019, with maximum summed concentrations of 446, and 2090 µg STX eq./kg respectively, with 2019 having an increased mean, median and range. On a group level the largest temporal variation (Figure 4) was between sessile fauna toxicity from 2018 to 2019, with low concentrations in 2018, but much higher toxicity in 2019, though this is possibly skewed, due to the increased sampling of the apparently more toxic species in 2019. Starfish and brittlestars, crustaceans (Anomura and Brachyura), molluscs and sea anemones (Actiniaria) all showed low concentrations of PST in both 2018 and 2019. Only one sunstar was sampled in 2018 making judgements on their temporal variability difficult, although it still showed toxicity (227 µg STX eq./kg). Although, the overall toxin concentrations seemed to increase from 2018 to 2019, PSTs at Station 1 appeared to decrease, with all nine samples analysed in 2018 PST-positive with an average toxicity of 131 µg STX eq./kg, whereas in 2019 only 25% of samples contained detectable levels of toxins with an average toxicity of 15 µg STX eq./kg. Spatial variability was notable (Figure 6 and Figure 7) with a ‘hot spot’ located at Station 2 in the Southern Bight of the North Sea. The mean toxicity at this sampling site was 429 ± 438 µg STX eq./kg (n = 8), which was more than double the toxicity of all other stations tested across both years, showing a total PST range of 88–1461 µg STX eq./kg. All samples analysed from Station 2 contained PSTs, regardless of species. Although, this location was only sampled in 2019. Station 3 saw the highest toxicity in a sample of *Alyoniudium diaphanium*, statistically (Figure 6), this was an outlier with the highest other toxic sample at that station being a common shore crab (*Carcinus maenas*) sampled in 2018 and found to contain just 44 µg STX eq./kg. Station 7 showed a high range of toxicities in 2019, however the median was low, showing that most samples contained little or no detectable levels of toxins, with the results skewed by one highly toxic sunstar sample. Figure 7 summarises all starfish and brittlestar toxicity data from each station. Starfish and brittlestar toxin data were analysed separately as these species were sampled extensively (n = 50) from all stations, giving a far more robust data set. From these data, notably higher toxicities were evident in samples taken from Stations 1 and 2, in comparison to all other locations, giving good evidence that toxicity is potentially dependent in part on geographical location.

### 2.6. Statistical Analysis

An ANOVA was performed on all variables, which highlighted group (*p* = 0.012), station (0.00006), year (*p* = 0.03) and bottom salinity (*p* = 0.03) as having a statistical effect on toxicity at the 95% confidence level. The year having a statistical effect on toxicity could be confounded by the sampling of more potentially toxic species during 2019. There was no statistical effect of depth (*p* = 0.60), bottom temperature (*p* = 0.06), surface temperature (*p* = 0.92) or surface salinity (*p* = 0.69) on toxicity. A Principal Component Analysis (PCA) confirmed these results, in that group and location were positively associated with toxicity. However, there was no association found between the environmental variables. A linear mixed effect model was fitted with group as a fixed variable and station as a random variable, which highlighted sunstar toxicity as statistically different from other groups (*p* = 0.0021). A second linear mixed effect model was fitted with group as a random variable and station as a fixed variable. This analysis highlighted Station 2 toxicity as statistically different from other stations (*p* = 0.0009; with Station 8 also showing significantly lower toxicity against the remaining stations, *p* = 0.003). A Tukey’s multiple comparison of means test results confirmed the linear model’s hypothesis for both group and station analysis. These results add weight to the original analysis that indicated Station 2 to be a potential ‘hot spot’ of toxicity, and that sunstars showed a significantly higher level of toxicity to other groups.

## 3. Discussion

### 3.1. Occurrence of Toxins

The vast majority of PST occurrence data generated globally relates to the presence of toxins in bivalve mollusc shellfish harvested from designated shellfish harvesting areas within inshore marine waters, as monitored under regulatory surveillance programmes [7,63]. Consequently, toxin prevalence data has, to date, typically focussed on a restricted range of taxa, the majority of which are bivalve molluscs, with occasional reference to marine gastropods [52,64] and crustaceans [65,66,67,68]. While, the occurrence of PST in other marine invertebrates is less well-understood, there has been an increasing number of reports of PSTs in echinoderms, gastropods and barnacles [2,53,58,69]. Even less frequently, with the exception of offshore scallop harvests [70], findings of toxins have been reported in more offshore benthic samples. Consequently, there is little information regarding the potential uptake, presence, or depuration of toxins from offshore, non-bivalve invertebrate fauna. The data generated in this study demonstrate, for the first time, extensive PST accumulation in the marine benthos across a large range of taxonomic groups across a geographical range from the Southern Bight to the Shetland Islands.

Four of the samples analysed (two colonies of *A. diaphanum* and two specimens of *C. papposus*) contained total summed PSTs above the EU MPL, highlighting some benthic species are capable of accumulating PSTs to dangerous levels if consumed by mammals. Notable toxicity above 200 µg STX eq./kg was also discovered in sea mouse, shrimp, common starfish, green sea urchin and brittlestar. Consequently, to the authors best knowledge, this is the first detection of PSTs in the phylum bryozoan (*A. diaphanum*), as well as eleven other new vectors (>50 µg STX eq./kg), specifically the gastropod *Scaphander lignarius*, the starfish *Anseropoda placenta*, *Asterias rubens*, *Astropecten irregularis*, *Luidia ciliaris* and *Stichastrella rosea*, the brittlestar *Ophiura ophiura*, the crustaceans *Atelecyclus rotundatus* and *Munida rugosa*, the sea mouse *Aphrodita aculeata*, and the sea urchin *Psammechinus miliaris*.

While, there is evidence for the presence of PSTs in the benthos, the primary source is still unknown. The global literature has mostly been able to link toxicity in marine invertebrates to the presence of blooms of known PST producers and subsequent trophic transfer. In this case, there is no evidence of a causative algal bloom at the time of sampling, across any of the sampling regions in either year. In this study, toxin content was geographically widespread, and ANOVA and PCA analysis confirmed a lack of correlation between toxicity and oceanographic factors, such as depth, temperature and salinity. Many organisms are known to graze on benthic cyanobacterial mats and so this could represent a source for toxins in organisms encountering these cyanobacterial proliferations if they were toxic. While, intertidal areas in the North Sea are known to experience cyanobacterial growth [16], there is little evidence to suggest that they proliferate at depth in temperate, deep, marine waters. In this study, as the majority of samples originated from deeper waters and not from coastal or transitional waters, there is no evidence to support benthic cyanobacterial mats as a source of saxitoxins in the contaminations presented, herein. However, it represents a potential source especially in fringing marine waters. Two other plausible causes of PST accumulation in the benthos, include the bioaccumulation of toxins from sedimentary algal cysts and/or the presence of PST producing bacteria, possibly in symbiosis with one or multiple benthic organisms. *Alexandrium* cyst populations can be present for hundreds of miles alongshore [24,71] and the toxicity in cysts can be comparable or more than their vegetative counterparts [72]. The presence of an ‘algal cyst bed’ would mean that the marine benthic fauna is potentially exposed to a highly toxic source, which could accumulate through a wide range of taxa, explaining the presence in all groups tested. There is evidence that once *Alexandrium* cysts reach the sea floor, the anaerobic and low light conditions can prevent germination indefinitely [73]. The cysts remain viable for many years may explain the presence of PSTs in offshore organisms, whilst inshore shellfish beds along the eastern English coast, have exhibited no evidence of toxicity in recent years [43]. Historically the south east of Scotland and north east of England have experienced PST outbreaks, with toxicity detected in shellfish regularly from 1968 to 1990 [65,74]. Later, sporadic *Alexandrium* algal cyst deposits from Aberdeen to Bridlington were discovered, with toxicity found in shellfish and crabs [75,76]. Since 2015, routine monitoring of phytoplankton detected seven sporadic occurrences of *Alexandrium* sp. along the east coast of the UK. Five of these were in 2018 (data available from [77]). It should be noted that these sampling points are nearshore, long distances from the offshore sampling locations used in this study. It is possible that these events, both historically and in 2018, could have seeded the benthos around that region with *Alexandrium* cysts. There is, however, no known domestic algal PST producer that exhibits a dcSTX profile and any cyst bed would have to stretch for hundreds of miles. Bacterial production of PSTs has been described previously [78] with microbiological symbiosis attributed with the accumulation of the neurotoxin TTX in marine life, specifically pufferfish [79] and the starfish *Astropecten polyacanthus* [80]. A symbiotic bacterial source could explain widespread toxicity if the causative bacteria are present in more than one organism and in multiple geographic locations. Additionally, it could explain why some organisms appear to accumulate more PSTs than others and why toxicity appears across many taxonomic groups. To elucidate the source of PSTs, extensive cyst bed analysis and microbiological screening of live organisms will be required.

### 3.2. Method Comparison

As the aim of this study was to examine the presence of a potentially novel toxin source with an unusual toxin profile in various un-validated matrices, it was important to utilise more than one detection method, in order to provide a higher level of certainty to any results generated. The results generated by LC-FLD and LC-MS/MS on the study samples showed the two methods performed similarly. The LC-MS/MS, however, produced a 15% positive bias vs the LC-FLD method, in terms of total PST concentrations, which was perhaps unexpected given the lack of chromatographic separation for epimeric pairs by LC-FLD, requiring the assumption that each pair exclusively contains the most toxic epimer, leading to over estimation [81,82]. This is a contributing factor in the samples from Stations 7 and 13 where GTX2 and 3 were present. Additionally, the differences are also likely due to the inclusion of a greater number of toxin analogues, specifically doSTX and dcGTX1, in the LC-MS/MS method and were present in 14 and eight samples respectively. Overall, the two methods compared well, providing confidence in the quantitated concentrations of PSTs reported in a large range of marine benthic organisms. Both the LC-FLD and LC-MS/MS methods have subsequently been validated for gastropods and crustaceans, with results reported elsewhere [83].

### 3.3. Toxin Profiles

Two dominant toxin profiles were identified following the cluster analysis of the quantitative data, one centred around dcSTX and the other containing high proportions of STX. The two profiles appear to be associated with specific locations, with the dcSTX dominant profile associated with samples in the south (Stations 1–3) and north of the study area (Stations 71, 73, 77), with the STX profile present in organisms harvested in more central regions (Stations 7 and 13). This suggests that the toxin profile in the benthos is linked to geographic location, rather than being related to the species of the contaminated organism. The high dcSTX profile is unusual, potentially resulting from enzymatic biotransformation, more specifically the potential action of carbomylase [46] and/or sulfocarbamolyase [47], across the sampled regions other than Stations 7 and 13. Enzymatic hydrolysis of PSTs into decarbamoyl variants has previously been reported in shellfish [44,46,47,84,85,86]. It is described as a species-specific transformation, only in a small number of clam species, so it is unlikely that such transformation is occurring in every species across multiple taxonomic groups, over wide geographic fetches, unless driven by bacteria [48,78,87,88] or other unknown means, which are only present in specific locations under certain conditions. In addition to enzymatic decarbamoylation, selective toxin retention or elimination, reductive conversion and hydrolysis are also known to affect toxin profiles in shellfish tissues [89,90,91]. Selective retention/elimination is highly unlikely here, given the extremely low relative proportions of dcSTX present in toxin-producing *Alexandrium* species found in the UK. Consequently, without the presence of enzymatic biotransformation, there is the potential for decarbamoylation to be triggered by other mechanisms relating to the conditions within the benthos at the bottom of the North Sea at depths of 30 m–170 m. Previous work has also highlighted varying toxin profiles in marine invertebrates in different geographical locations [54,58]. Silva et al. 2018 [58] focussed on three geographical locations with each group of samples showing high proportions of the decarbomyl toxins dcGTX2&3, whilst Silva et al. 2013 [54] described high dcSTX content in some gastropods and bivalves, which could evidence enzymatic change in those environments. In both studies, profiles varied greatly between species, whereas conversely results reported here show consistent profiles based on location, regardless of taxonomic group. Alternatively, the toxin profiles measured in the benthos here may be similar to the toxin profiles within the primary producers, as seen in some shellfish [89,92]. To date, no phytoplankton species, detected in UK waters, have been found to produce any significant levels of decarbamoyl PST analogues. In the absence of both a dcSTX-producing *Alexandrium* sp. and toxin transformation pathways within the benthos tissues, a potentially novel source of PSTs may be considered. Samples from Stations 7 and 13 contained high proportions of STX profile and GTX2, and the profile was more representative of the profile reported in bivalve molluscs along English and Scottish coasts [43]. Both these stations were close to the extensive coastal cyst beds discovered from 1995 and 1997 [75,76], suggesting *Alexandrium* cysts as the potential source in these locations. These results could therefore indicate the possibility of two different toxin sources in the benthos, one unknown producing a dcSTX profile and a conventional domestic algal cyst bed producing the STX and GTX 2 profile. Without extensive and widespread sediment analysis, it is impossible to definitively state the source of the PSTs or the reason for the differences in toxin profile.

### 3.4. Group Variability

The data presented here highlights large variability in PST concentrations determined between different benthic groups. Due to the non-targeted nature of the sampling, drawing conclusions on inter-group toxicity is difficult, given that geographic location is also an important factor influencing toxicity, as exemplified by the highly toxic samples from Stations 2–3 and the absence of detectable PST at Station 71 (a linear mixed effect model highlighted Station 2 as having significantly different toxicity from all other stations). Without identifying the primary source of PSTs, and identifying transfer mechanics through the benthos, it is currently impossible to determine the cause(s) of inter-group variability. From these data, however, a variety of organisms in the benthos have accumulated PSTs, highlighting widespread exposure to a PST producer, most notably sunstars and *A. diaphanum* appearing capable of accumulating high toxin concentrations.

Of the sessile fauna tested, only the bryozoan *A. diaphanum* contained PSTs above detectable levels. It exhibited the highest toxicity of any sample tested (2091 µg STX eq./kg), two of which were above the EU MPL. They exhibited a mean toxicity of 926 µg STX eq./kg (n = 4, all from 2019), which is higher than sunstar. Bryozoans are filter feeders and responsible for producing a wide range of chemical metabolites [93]. *Alcyonidium diaphanum* produces a sulfoxonium ion which causes the dermatitis condition ‘Dogger Bank Itch’ [94]. Due to the high toxicities discovered and its relative abundance in British waters [95], it is plausible that *A. diaphanum* plays an important role in the occurrence and transfer of PSTs in the benthos. As *A. diaphanum* are filter feeders, it is possible that PST accumulation in these organisms are the result of algal cyst ingestion, and that subsequent accumulation in higher trophic organisms is due to predation on *A. diaphanum.* However, *A. diaphanum* only had high levels of PSTs in the more toxic locations (Stations 2–3), with low toxicities at Station 71, highlighting a similar location-driven toxicity as found for other species, thus potentially ruling it out as the route of PSTs into the benthos. As only four samples were analysed from three locations, all in 2019, the data set is too small to make any conclusions on its spatial and temporal variability, however, their ability to accumulate PSTs is clear.

Sunstar toxicity determined here was lower than the levels of toxicity reported in samples associated with the canine intoxications [42]. However, all sunstars analysed were ubiquitously toxic regardless of location or year. In 2019, the sunstar from Station 7 was toxic (1275 µg STX eq./kg), whereas all other organisms tested from Station 7 had no PSTs detected or showed only trace concentrations. Consequently, this provides some evidence for sunstar toxicity being independent from location, which is different to all other groups tested. The results from the linear mixed model support this hypothesis, which highlighted sunstars as having significantly different toxicity to the other groups analysed.

The mechanisms for sunstar toxin presence have yet to be elucidated, with further work involving live organisms required to generate supportive data. The organisms could feasibly accumulate PSTs from a dietary source, which has been used to explain echinoderm toxicity before [38,53]. In those cases, starfish toxicity was linked to starfish predation on a highly toxic bivalve food source. Given that scallops are commonly found in deeper offshore waters and are capable of accumulating PSTs [70], this could be a route of trophic transfer into sunstars. In this study, there was no evidence to confirm such trophic transfer, given the limited bivalve mollusc samples. However, the accumulation of toxins from a dietary source is unlikely as the most toxic organisms analysed were from a range of feeding guilds (Table 1), with high concentrations observed in both scavenging-predators (*C. papposus*) and filter-feeders (*A. diaphanum*). More intensive sampling of benthic invertebrates at specified locations would be required to better understand how toxin concentrations may be influenced by feeding guild and other factors, such as the relationship to the sediment, which may influence interactions with algal cysts or toxic organisms. Additionally, sunstars are unlikely to have vastly different diets to some other starfish species (e.g., *Asterias rubens*), highlighting starfish and sunstars utilising the same niche showed significantly different toxicities. Sunstars have also been shown to be adaptive hunters, preying on readily available organisms [96], thus, making it unlikely that their prey are the same in all the geographic locations. Although, toxin accumulation in sunstars could feasibly occur following ingestion of algal cysts, it is unclear why any potential accumulation via this route is far more consistent in sunstars than other benthic organisms. Sunstars could have a low toxin depuration rate, as noted in abalone gastropods [97,98,99], and which could explain consistently high toxicity, potentially making them more at risk than other benthic organisms for accumulating PSTs. This would imply that larger and thus older sunstars [96] should have higher toxicity. The study showed no correlation between diameter of sunstar and toxicity (n = 6) (data not shown). For cysts to be the source, sunstars would need to have an active storage mechanism and any cyst deposits would have to be geographically extensive and composed of cysts from different algal species or strains to give rise to the different PST profiles observed within this study. The potential for primary production of PSTs by sunstars is possible, due to a microbial symbiosis similar to that of TTX presence in pufferfish [79]. The presence of TTX producing vibrios has previously been noted in the starfish *Astropecten polyacanthus* [80]. A symbiotic bacterial source of PSTs could explain the consistent and widespread toxicity in sunstars, which had a statistically different toxicity to all other groups analysed. Ultimately, sunstars acting solely as a primary producer remains unproven noting that sunstars from this study also exhibited the same location driven profile variation as all other groups. Future work, involving sunstars from a range of geographical locations, is required in order to begin to understand the presence and potential accumulation and depuration of PSTs in starfish. Without an extensive uptake, depuration and elimination study, and a full molecular analysis of any associated bacterial fauna, drawing conclusions on whether sunstar toxicity is acquired or produced is currently impossible.

### 3.5. Spatial and Temporal Variability

Results appear to highlight geographic location as an important factor in toxicity, with high toxicities above 400 µg STX eq./kg at Stations 1–3, 7. The highest toxicities in non-sunstars were from Stations 1–3, all located in the Southern Bight. Trace or non-detectable toxicities were found in Stations 4, 8 and 43, which were all further offshore, although ANOVA, PCA and mixed model analysis showed no statistical link between distance from shore and toxicity. Station 73 seemed to exhibit high toxicity, however, only one sample (a sunstar) was analysed, which skewed the analysis. The identification of a potential ‘hot spot’ at Station 2 was important, showing higher mean toxicities than other stations and statistical differences compared to other locations, noting no sunstars were sampled from this station. Figure 7 illustrates the spatial variability between stations, as the data set is far more robust, given starfish and brittlestars were widely sampled and analysed from most stations. The results showed the highest toxicities in samples from Station 2, with notable toxicity in Station 1 and trace toxicities everywhere else. The location of this hotspot is unexpected given previous reports of a cyst bed and historical PSP events around the region close to Stations 7 and 13. Data from this study shows that samples from these sites are generally low in toxicity, albeit with high variability. Although, toxicity was widespread along the East coast of the UK, both sunstars [100] and *A. diaphanum* [95] are common in the Bristol Channel and Irish Sea, possibly indicating those areas could also be at risk of exhibiting PSTs in the benthos.

In terms of temporal variability, total toxin concentrations appeared to be higher in samples taken from 2019 in comparison to those sampled during 2018 (Figure 5). However, higher mean toxicities could be a result of more consistently toxic species being sampled, for example, the higher number of sunstars and *A. diaphanum* sampled in 2019, or by the sampling at a more toxic location, such as Station 2 which was only sampled in 2019. Further analysis in future years is required to determine whether Station 2 remains a region associated with highly toxic benthic organisms over time. Samples taken from Station 1 showed a notable decrease in toxicity from 2018 to 2019, with all 2018 samples showing detectable levels of toxins, with a maximum of 446 µg STX eq./kg, as opposed to samples from 2019 where 75% of samples had no PSTs detected with a maximum of only 17 µg STX eq./kg. This represents a large difference between years and indicates that the source that was present in 2018 had either moved or reduced significantly. Given the notable changes in toxin content between the two years, there may be potential differences from current-related movement of benthos and/or cyst beds from site to site over time [101,102]. However, more work is required over a larger number of years and in a higher number of geographical regions to enable any such assessments to be made.

### 3.6. One Health Considerations

Results from this study provide strong evidence for the accumulation of PST in a large range of benthic species over a wide geographical area within the North Sea. Four samples were found to contain total toxin concentrations exceeding the EU MPL of 800 µg STX eq./kg. The implications of these findings in relation to the risks to consumers of seafood originating from the North Sea is unclear. The most notable human food stuff analysed were shrimps with the highest toxicity determined in shrimp of 445 µg STX eq/kg from Station 1 in 2018. This evidences toxicity in shrimps following toxin accumulation, as previously reported in samples of penaeid shrimp from Brunei and Malaysia (reviewed in [2]), inferring at least some level of human intoxication risk from ingestion of shrimp caught from certain areas. While, crabs are also a commonly consumed food source, all the crabs analysed in this study were small, mostly non-edible species, with the exception of *Necora puber*. Numerous reports exist for PST presence in a range of crab samples such as *Cancer* sp., *Fabia* sp., *Hemigrapsus* sp., *Pugettia* sp., *Portunus* sp., *Pilumnus* sp., *Metograpsus* sp. and *Telmessus* sp. [2]. Most notably, extreme levels of toxicity have been reported in some species of xanthid crabs where PSP has been measured at concentrations far above the MPL [103]. Trophic transfer of PSTs into commercially-important species, specifically edible crab (*Cancer pagurus*) and lobster (*Homarus gammarus*) is a potential unregulated intoxication route to humans, as both these species are extensively fished within the inshore waters along the eastern and southern coasts of the UK [104]. Edible crabs in particular are potentially co-inhabiting the benthic environment with the toxic benthos, and are known scavengers that also feed on a wide range of prey [105,106,107,108]. Trophic transfer of PSTs into crab species is possible [50,109] and capable of accumulating to levels above the regulatory limit [69], posing the risk that edible crabs species in northern European seas are potentially under risk of accumulating PSTs.

Monitoring PSTs in marine benthos has several drawbacks versus bivalves, primarily that many invertebrates are motile, there are no validated detection methods for most non-bivalve species and uptake and depuration kinetics are un-characterised [69]. As no edible crabs were sampled during the study, the risks are still unclear. Future work is essential to assess the risk in edible crabs and other commercially important, but unregulated vectors.

There is also potential risk to the health of other marine organisms, with high concentrations of toxins accumulating in several benthic species. Benthic toxicity at levels above the EU MPL may have detrimental effects to other species. With some species, such as xanthid crabs and sunstars, known to accumulate high concentrations, trophic transfer and subsequent bioaccumulation could impact upon animal health of higher level predators, such as lobsters and larger crabs [110]. There are reports of some crab species producing the protein saxiphilin which has been postulated as explaining STX-resistance to toxic effects and hence the ability to tolerate toxicity within their flesh [111]. With some organisms known to retain toxicity for long periods of time, risks may be present for a period of many years [112,113].

The presence of toxins in benthic invertebrates that may be washed ashore has societal implications, with beach scavenged crabs and starfish implicated in recent canine deaths [42]. Anecdotally *A. diaphanum* was found among the stomach contents of a dog that had died following a beach walk in 2005 (pers.comm [114]) and implicated in multiple dog deaths in 2006 [115], however, in neither case were PSTs tested for and no archived samples were stored for retrospective analysis. Consequently, further studies on the spatial, temporal and taxonomic patterns in toxins in benthic invertebrates, especially those that are known to be washed ashore after periods of disturbance, could usefully be undertaken. Also important is determining likely levels of risk to the wider animal and ecosystem health, and therefore ‘One Health’ within the benthic marine environment when exposed to high levels of PST.

## 4. Conclusions

Analysis of a wide range of benthic marine organisms, sampled over a two-year period from multiple sites within the North Sea, revealed the unexpected presence of PSTs in offshore, benthic environments. PST accumulation was geographically extensive, capable of accumulating to dangerous levels in certain species and was associated with two separate toxin profiles, potentially inferring either more than one toxin source and/or extensive toxin transformation. During the study, 12 new vectors of PSTs were identified, with the sunstar *C. papposus* and the bryozoan *A. diaphanum* emerging as key species. Sunstars appeared to always possess PSTs and showed statistically higher toxicity than other taxonomic groups, possibly highlighting that they either produce PSTs or can accumulate and store them. Three sampling stations from the Southern Bight showed high toxin concentrations in sampled species, with data showing a statistical difference from samples taken from other parts of the coast. Currently the ‘One Health’ risks remain unclear. While, some of the edible organisms were found to be toxic, the likelihood of accumulation in commercially important stocks would need to be assessed. Similarly, the potential threat to animal and ecosystem health needs further investigation. The toxin source of PSTs is also currently unclear, which outlines key areas of future work required. This includes further analysis of benthic organisms from other at risk locations, a higher spatial resolution of toxicity in the Southern Bight and eastern English Channel, sediment analysis of the East coast of the UK, uptake, toxin conversion and elimination studies of PSTs in sunstars, and analysis of their microbiological fauna. Ultimately, this work will be able to aid risk managers to better understand the risk to commercially important, but currently unregulated, foodstuffs, as well as any potential risks to animal and ecosystem health.

## 5. Materials and Methods

### 5.1. Sample Collection Methods

Samples were collected during the English International Bottom Trawl Survey (IBTS-Q3) conducted during August 2018 and 2019. These surveys targeted specific locations around the North Sea where a GOV (Grand Overture Verticale) otter trawl net was deployed, configured to IBTS-Q3 series standard, including 20 mm cod-end liner. Fishing was conducted for 30 min at a speed of 4 knots. Examples of benthos caught were retained and frozen onboard the vessel from multiple fixed stations (Figure 4), identified before the surveys. Once the surveys were completed, the frozen samples were transported to Cefas where they were held in frozen storage until required for analysis.

### 5.2. Samples

Across the two years, twelve sampling locations were assessed, ranging from the Southern Bight to the north of the Shetland islands (Figure 4). Station locations 1, 3, 4, 7, 13, 22 and 77 were sampled in both years, whereas Station 8 was only sampled in 2018 and Stations 2, 43, 50, 71 and 73 were only sampled during 2019. In total, 167 samples were collected and analysed, with 64 from 2018 and 104 from 2019. Once samples were received, organisms were identified visually to species level where possible, however, where this was not possible identification to genus level was acceptable. In the case of some sessile fauna samples, only identification to phylum level was possible. Over 30 distinct benthic species were recorded. Additional station information can be found in Table A2 in the Appendix A.

### 5.3. Reagents and Chemicals

Certified reference toxins were obtained from the Institute of Biotoxin Metrology, National Research Council Canada (NRCC, Halifax, NS, Canada). Toxins incorporated included GTX1-6, dcGTX2&3, dcSTX, dcNEO, NEO, STX and C1&2. Non-certified toxin standards were also received from CNC (Nelson, New Zealand) for C3&4 and dcGTX1&4. LC-MS grade water was produced by a MilliQ water purification system (Merck, Darmstadt, Germany). All solvents, reagents and chemicals were of LC-MS or HPLC grade, depending on the system specific requirements.

### 5.4. Sample Preparation and Extraction

Benthic organisms were assessed visually from each sampling point. When more than one organism of the same species was present in a specific location, individuals were all pooled and taken as a single representative sample. This was performed for all species except sunstars (*C. papposus*). In order to estimate whether toxicity of sunstars correlated with diameter, each sunstar was analysed separately. All samples were subsequently homogenised using Waring industrial blenders (Stamford, Connecticut, USA) and IKA Ultra Turrax homogenisers (Oxford, Oxfordshire, UK).

Samples collected in 2018 were extracted using two different methods, samples analysed utilising ultra-high-performance liquid chromatography with tandem mass spectrometry (LC-MS/MS) required a 5 g aliquot to be extracted using 5 mL 1% Acetic acid, using a single step dispersive extraction [116]. Where possible a 1:1 sample to solvent ratio was used. Samples analysed utilising pre-column oxidation liquid chromatography with fluorescence detection (LC–FLD) were extracted using a two-step exhaustive extraction [117], with 5 g aliquots extracted twice each using 3 mL 1% Acetic acid. For small samples where 5 g was not available, a scaled down extraction was used, with absolute amounts depending on the volume of homogenised tissue available. For samples where insufficient material was collected to perform both extractions, analysis by LC-MS/MS was prioritised due to wider range of quantified toxins incorporated. For samples collected during 2019, a unified extraction was performed to allow both methods to be applied to all samples. For these samples, a refined version of the LC-MS/MS extraction method was conducted, incorporating a higher solvent to sample ratio, specifically 2 g tissue plus 18 mL 1% Acetic acid.

Graphite solid phase extraction (SPE) clean-up was conducted to remove salts from the acidic extract [118], before dilution of SPE eluant with acetonitrile and LC-MS/MS analysis. For samples destined for LC-FLD, crude acidic extracts were subjected to C18 SPE clean up, followed by pH adjustment to 6 ± 1 and dilution to volume. Quantitation was achieved following the LC-FLD analysis of peroxide-oxidised C18 SPE-cleaned extracts and analysis of an un-oxidised extract to identify any naturally fluorescent co-extractives [117]. Due to the laborious nature of fully quantifying samples by LC-FLD and the expected high dcSTX and STX profile, a semi quantitative screen was initially performed to identify samples that contained any N-hydroxylated compounds, which if present were forwarded for ion exchange SPE and periodate oxidation of isolated fractions.

### 5.5. Analysis of PSTs

**Figure 8:**
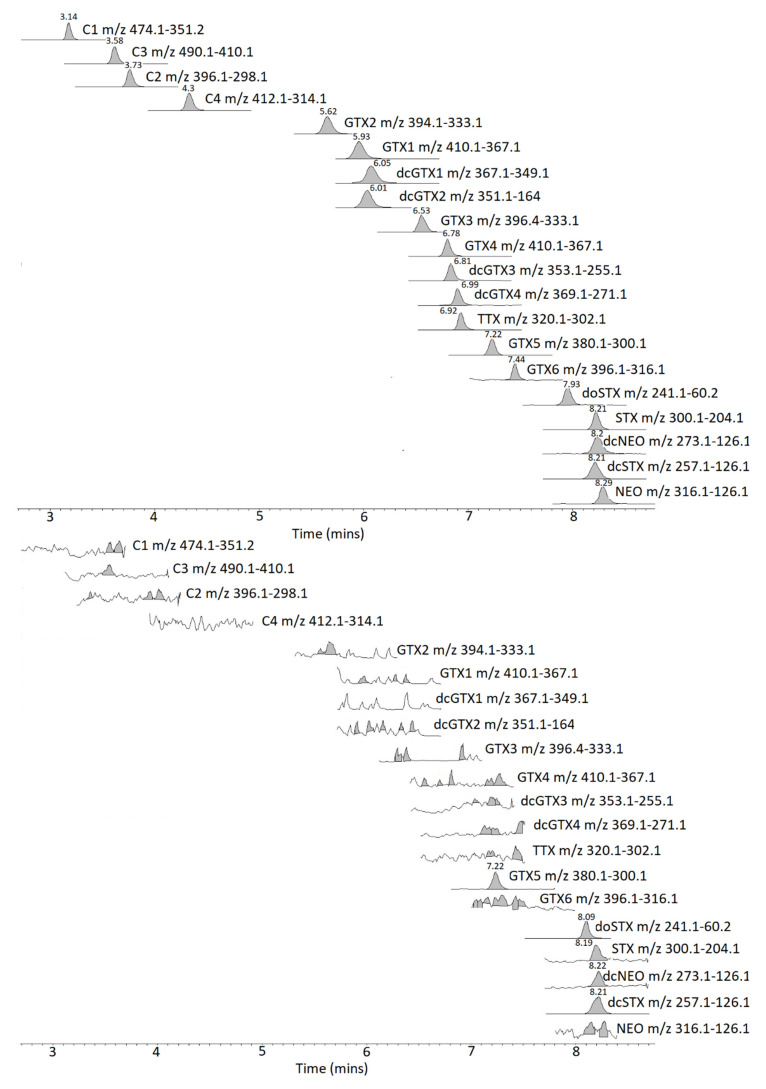
Chromatogram detailing LC-MS/MS quantitative m/z transitions for certified standards (top) and a positive Sunstar (CEND181) (bottom).

**Figure 9:**
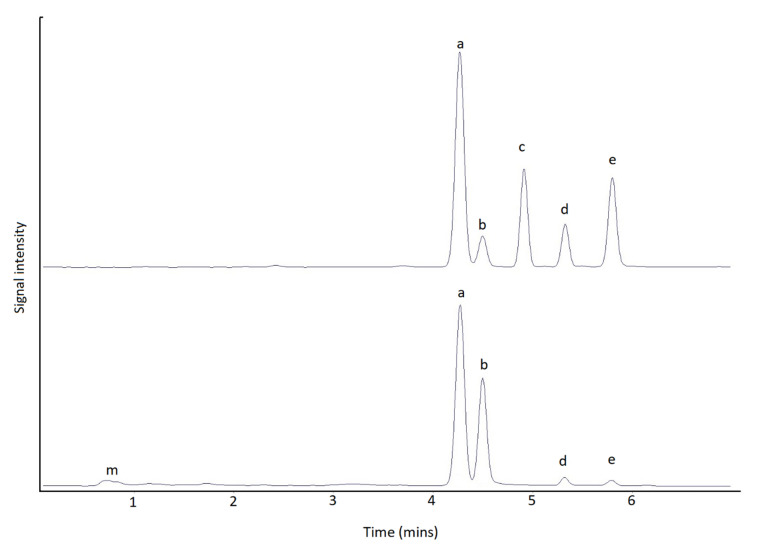
Chromatogram of LC-FLD for certified standards (top) and positive Sunstar (CEND181) (bottom). Key a—dcSTX quantitative, b—dcSTX qualitative, c—GTX2&3, d—GTX 5, e—STX, m—matrix.

LC-MS/MS analysis was performed using an Agilent (Manchester, UK) 6495B triple quadrupole tandem mass spectrometer, with chromatography conducted using an Agilent 1290 Infinity II UHPLC system. Chromatographic separation was achieved using either an Agilent Poroshell 120 HILICZ (150 mm × 2.1 mm × 2.7 µM) or a Waters Acquity BEH Amide (150 mm × 2.1 mm × 1.7 µM) (Elstree, Herefordshire, UK) column utilising a gradient solvent delivery. All instrument and chromatographic criteria are as described in [116]. An analysis of each toxin analogue was carried out using two multiple reaction monitoring (MRM) transitions, as detailed in [116], with quantitation performed using a six point calibration curve for each primary transition prepared using certified calibrants diluted in PST negative SPE-cleaned and diluted mussel extract. The LC-MS/MS method was validated previously for the quantification of GTX1-6, dcGTX1-4, C1-4, doSTX, dcSTX, dcNEO, NEO, STX in molluscs as well as the bacterially-derived neurotoxin Tetrodotoxin (TTX). Chromatograms of certified standards and a positive sunstar are detailed in Figure 8. LC-FLD analysis was performed on an Agilent 1200 LC system consisting of a quaternary pump, FLD, vacuum de-gasser, autosampler and thermostatically controlled column oven. Chromatographic separation was achieved using an Phenomenex Kinetex C18 (150 mm × 4.6 mm × 5 µM) (Torrance, CA, USA) column, adopting a solvent gradient as detailed in [119]. Quantitation of oxidized PSTs was achieved using a six-point calibration curve, which was prepared using certified calibrants diluted in 0.01M HAC. The LC-FLD method quantified the epimeric pairs, GTX1 and 4, GTX2 and 3, C1 and 2, C3 and 4 and dcGTX2 and 3, as well as the analogues GTX5, GTX6, NEO, dcNEO, dcSTX and STX. Chromatograms of certified standards and a positive sunstar are detailed in Figure 9.

### 5.6. Data Analysis

Toxin profiles were analysed using a K-means clustering algorithm [61], which assigns statistical centers based upon toxin content (based on each toxin as a percentage of total toxicity, expressed in µg STX eq./kg) of samples and then ‘sorts’ samples into clusters based upon the statistical distance from each center. This approach was previously used for toxin profile analysis in [43]. A 95% confidence paired students t-test was used to analyse statistical differences of samples >80 µg STX eq./kg between the LC-FLD and LC-MS methods. An analysis of means and standard deviations and creation of box plots of PST concentrations only used samples which detected PSTs above LOD, all samples <LOD were removed from the analysis. Prior to the statistical analysis, the toxicity variable was log transformed. An analysis of variance (ANOVA) was performed to investigate the most influencing variables on the toxicity. Normality of the residuals was checked. A Principal Component Analysis was then conducted to explore data variation among observations described by a mixture of qualitative and quantitative variables. The interaction between the most influential variables was analysed by comparing nested models including interaction terms or additive terms only [119]. Eventually, linear mixed effect models fitted to the data and followed by a Tukey’s multiple comparison were used to estimate differences in toxicity between groups, and locations, respectively. All statistical analyses were performed using R statistical software [120], and packages *PCAmixdata* [121], *nlme* [122] and *multcomp* [123].

## Figures and Tables

**Table 1:** Summary of groups, associated species and mean total toxicities (µg STX eq./kg) and total toxicity ranges (µg STX eq./kg) for all benthic organisms sampled.

Faunal Group	Species	Feeding Guild	Mean Toxicity	s.d	Toxicity Range	Number of Samples	% Samples > LOD
Sunstar	*Crossaster papposus*	SP	448	472	98–1275	7	100%
Starfish & Brittlestar (excluding *C. papposus*)	*Anseropoda placenta, Asterias rubens, Astropecten irregularis, Henricia oculata, Henricia* sp., *Hippasteria phrygiana, Luidia ciliaris, Porania pulvillus, Stichastrella rosea, Ophiura ophiura*	SP	80	126	nd–488	50	56%
Sea urchins	*Echinus esculentus, Echinus* sp., *Psammechinus miliaris*	OG	75	122	nd–257	11	36%
Crustaceans (Natantia and Stomatopoda)	*Rissoides desmarseti*	SP	161	247	nd–446	6	50%
	*Crangon* sp., *Pandalus* sp., unidentified Natantia	DS/SP					
Crustaceans (Anomura and Brachyura)	*Atelecyclus rotundatus, Atelecyclus* sp., *Carcinus maenas, Corystes cassivelaunus, Inachus* sp., *Liocarcinus depurator, Liocarcinus holsatus, Liocarcinus* sp., *Munida rugosa, Necora puber, Pagurus bernhardus, Pagurus* sp., Portunidae (indet.)	SP	25	22	nd–88	37	70%
	*Goneplax rhomboides*	DS					
Polychaetes	*Aphrodita aculeata*	SP	182	175	nd–386	13	54%
Molluscs	*Aequipecten opercularis*, Bivalvia (indet.)	FF	66	51	nd–172	17	65%
	*Crepidula fornicata*	FF/OG					
	*Hinia reticulata, Scaphander lignarius*	DS					
	Buccinidae (indet.), *Buccinum undatum, Colus gracilus*	DS/SP					
	*Neptunea antiqua* eggmass	N/A					
Actiniaria	*Metridium senile*	FF	40	44	nd–150	11	82%
	Anemone (indet.)	P					
Sessile colonial fauna	*Alcyonidium diaphanum, Alcyonium digitatum, Haliclona oculata*, Porifera (indet.)	FF	488	818	nd–2090	14	57%
Other	*Parastichopus tremulus*, Echiura (indet.)	DF	nd		nd	2	0%
Total			127	301	nd–2090	168	61%

nd: not detected. Feeding guilds for benthic invertebrates were assumed to be comprised of one of the following seven categories: filter- and suspension feeders (FF), algal grazers (AG, not sampled in present study), omnivorous grazers (OG), deposit feeders (DF), detritivores/scavengers (DS), scavengers/predators (SP) and facultative predators (P). Feeding guilds adapted from [59,60].

**Table 2:** Comparison of results between samples analysed by the LC-FLD and LC-MS/MS methods.

Criteria	LC-FLD	LC-MS/MS
Mean toxicity (µg STX eq./kg)	130	145
Sd	366	334
Maximum toxicity (µg STX eq./kg)	2744	2091
Correlation	0.87	
t score	−0.47	
t crit	2	
Means tested	15%	

## Appendix A

**Table A1:** Full table of all samples analysed from both years, with total toxicities in µg STX eq./kg, species and taxanomic groups. nd; not detected, nt; not tested.

Cefas ID	Year	Station	Common Name	Species	Group	Total PST Detected by LC-FLD ( µg STX eq./kg)	Total PST Detected by LC-MS/MS ( µg STX eq./kg)
CEND 001	2018	22	Rigid cushion starfish	*Hippasteria phrygiana*	Starfish & Brittlestars (excl Sunstar)	9	4
CEND 002	2018	22	Whelk	Buccinidae (indet.)	Molluscs	27	70
CEND 003	2018	22	Woody canoe bubble	*Scaphander lignarius*	Molluscs	nt	102
CEND 004	2018	22	Sandstar	*Astropecten irregularis*	Starfish & Brittlestars (excl Sunstar)	13	66
CEND 005	2018	22	Hermit crab	*Pagurus bernhardus*	Crustacean (Anomura & Brachyura)	nt	nt
CEND 006	2018	22	Common starfish	*Asterias rubens*	Starfish & Brittlestars (excl Sunstar)	34	65
CEND 007	2018	22	Circular crab	*Atelecyclus rotundatus*	Crustacean (Anomura & Brachyura)	4	54
CEND 008	2018	22	Sea mouse	*Aphrodita aculeata*	Polychaeta	25	359
CEND 009	2018	13	Anemone	Actiniaria (indet.)	Actiniaria	nt	5
CEND 010	2018	13	Dead-man’s fingers	*Alcyonium digitatum*	Sessile colonial fauna	2	2
CEND 011	2018	13	Common starfish	*Asterias rubens*	Starfish & Brittlestars (excl Sunstar)	16	28
CEND 012	2018	13	Hermit crab	*Pagurus bernhardus*	Crustacean (Anomura & Brachyura)	nt	48
CEND 013	2018	13	Whelk	*Neptunea antiqua* (eggmass)	Molluscs	nt	5
CEND 014	2018	8	Masked crab	*Corystes cassivelaunus*	Crustacean (Anomura & Brachyura)	75	8
CEND 015	2018	8	Square crab	*Goneplax rhomboides*	Crustacean (Anomura & Brachyura)	14	8
CEND 016	2018	8	Swimming crabs	*Liocarcinus* sp.	Crustacean (Anomura & Brachyura)	4	13
CEND 017	2018	8	Common starfish	*Asterias rubens*	Starfish & Brittlestars (excl Sunstar)	6	2
CEND 018	2018	8	Dead-man’s fingers	*Alcyonium digitatum*	Sessile colonial fauna	nt	2
CEND 019	2018	77	Circular crab	*Atelecyclus rotundatus*	Crustacean (Anomura & Brachyura)	15	13
CEND 020	2018	77	Queen scallop	*Aequipecten opercularis*	Molluscs	41	103
CEND 021	2018	77	Swimming crabs	*Liocarcinus* sp.	Crustacean (Anomura & Brachyura)	8	14
CEND 022	2018	77	Brittlestar	*Ophiura ophiura*	Starfish & Brittlestars (excl Sunstar)	32	39
CEND 023	2018	77	Common starfish	*Asterias rubens*	Starfish & Brittlestars (excl Sunstar)	18	27
CEND 024	2018	77	Rigid cushion starfish	*Hippasteria phrygiana*	Starfish & Brittlestars (excl Sunstar)	21	23
CEND 025	2018	77	Seven-armed starfish	*Luidia ciliaris*	Starfish & Brittlestars (excl Sunstar)	nt	45
CEND 026	2018	77	Squat lobster	*Munida rugosa*	Crustacean (Anomura & Brachyura)	14	57
CEND 027	2018	77	Anemone	Actiniaria (indet.)	Actiniaria	nt	150
CEND 028	2018	77	Sea mouse	*Aphrodita aculeata*	Polychaeta	nt	98
CEND 029	2018	77	Hermit crab	*Pagurus bernhardus*	Crustacean (Anomura & Brachyura)	27	63
CEND 030	2018	77	Sandstar	*Astropecten irregularis*	Starfish & Brittlestars (excl Sunstar)	4	4
CEND 031	2018	3	Brittlestar	*Ophiura ophiura*	Starfish & Brittlestars (excl Sunstar)	nt	nd
CEND 032	2018	3	Shrimp	*Crangon* sp.	Crustacean (Natania & Stomatopoda)	nt	nd
CEND 033	2018	3	Swimming crabs	*Liocarcinus* sp.	Crustacean (Anomura & Brachyura)	nt	14
CEND 034	2018	3	Anemone	Actiniaria (indet.)	Actiniaria	5	12
CEND 035	2018	3	Common starfish	*Asterias rubens*	Starfish & Brittlestars (excl Sunstar)	nt	1
CEND 036	2018	3	Netted dog whelk	*Hinia reticulata*	Molluscs	nt	nd
CEND 037	2018	3	Common shore crab	*Carcinus maenas*	Crustacean (Anomura & Brachyura)	15	44
CEND 038	2018	4	Hermit crab	*Paguris bernhardus*	Crustacean (Anomura & Brachyura)	3	25
CEND 039	2018	4	Brittlestar	*Ophiura ophiura*	Starfish & Brittlestars (excl Sunstar)	nt	nd
CEND 040	2018	4	Common starfish	*Asterias rubens*	Starfish & Brittlestars (excl Sunstar)	3	nd
CEND 041	2018	4	Velvet swimming crab	*Necora puber*	Crustacean (Anomura & Brachyura)	13	7
CEND 042	2018	4	Sea urchin	*Echinus* sp.	Sea urchin	13	nd
CEND 043	2018	4	Sea urchin	*Echinus* sp.	Sea urchin	27	nd
CEND 044	2018	4	Swimming crabs	*Liocarcinus* sp.	Crustacean (Anomura & Brachyura)	47	16
CEND 045	2018	4	Circular crab	*Atelecyclus rotundatus*	Crustacean (Anomura & Brachyura)	188	15
CEND 046	2018	1	Shrimp	*Crangon* sp. *& Pandalus* sp.	Crustacean (Natania & Stomatopoda)	181	446
CEND 047	2018	1	Dead-man’s fingers	*Alcyonium digitatum*	Sessile colonial fauna	88	36
CEND 048	2018	1	Brittlestar	*Ophiura ophiura*	Starfish & Brittlestars (excl Sunstar)	nt	113
CEND 049	2018	1	Hermit crab	*Pagurus bernhardus*	Crustacean (Anomura & Brachyura)	nt	52
CEND 050	2018	1	Whelk	*Buccinum undatum*	Molluscs	nt	48
CEND 051	2018	1	Circular crab	*Atelecyclus rotundatus*	Crustacean (Anomura & Brachyura)	nt	7
CEND 052	2018	1	Slipper limpet	*Crepidula fornicata*	Molluscs	nt	172
CEND 053	2018	1	Green sea urchin	*Psammechinus miliaris*	Sea urchin	nt	23
CEND 054	2018	1	Sea mouse	*Aphrodita aculeata*	Polychaeta	nt	18
CEND 055	2018	1	Common starfish	*Asterias rubens*	Starfish & Brittlestars (excl Sunstar)	nt	395
CEND 056	2018	7	Common sea urchin	*Echinus esculentus*	Sea urchin	nt	2
CEND 057	2018	7	Bloody Henry starfish	*Henricia* sp.	Starfish & Brittlestars (excl Sunstar)	nt	15
CEND 058	2018	7	Common starfish	*Asterias rubens*	Starfish & Brittlestars (excl Sunstar)	33	11
CEND 059	2018	7	Seven-armed starfish	*Luidia ciliaris*	Starfish & Brittlestars (excl Sunstar)	26	72
CEND 060	2018	7	Swimming crabs	*Liocarcinus holsatus & Liocarcinus depurator*	Crustacean (Anomura & Brachyura)	73	11
CEND 061	2018	7	Dead-man’s fingers	*Alcyonium digitatum*	Sessile colonial fauna	nt	12
CEND 062	2018	7	Sunstar	*Crossaster papposus*	Sunstar	302	227
CEND 063	2018	7	Anemone	Actiniaria (indet.)	Actiniaria	nt	22
CEND 064	2018	7	Sea mouse	*Aphrodita aculeata*	Polychaeta	14	nd
CEND 065	2019	71	Common Starfish	*Asterias rubens*	Starfish & Brittlestars (excl Sunstar)	20	20
CEND 066	2019	71	Sea urchin	*Echinus* sp.	Sea urchin	10	nd
CEND 067	2019	71	Sea cucumber	*Parastichopus tremulus*	Other	9	nd
CEND 068	2019	71	Red Cushion Starfish	*Porania pulvillus*	Starfish & Brittlestars (excl Sunstar)	nd	nd
CEND 076	2019	71	Sunstar	*Crossaster papposus*	Sunstar	120	135
CEND 077	2019	71	Sunstar	*Crossaster papposus*	Sunstar	132	131
CEND 078	2019	71	Sunstar	*Crossaster papposus*	Sunstar	176	169
CEND 079	2019	71	Sunstar	*Crossaster papposus*	Sunstar	103	98
CEND 080	2019	71	Sponge	*Porifera* sp.	Sessile colonial fauna	7	nd
CEND 081	2019	71	Sea chervil	*Alcyonidium diaphanum*	Sessile colonial fauna	nd	9
CEND 082	2019	71	Anemone	Actiniaria (indet.)	Actiniaria	9	nd
CEND 083	2019	71	Woody canoe bubble	*Scaphander lignarius*	Molluscs	nd	nd
CEND 084	2019	71	Seven-armed Starfish	*Luidia ciliaris*	Starfish & Brittlestars (excl Sunstar)	nd	nd
CEND 085	2019	71	Rosy Starfish	*Stichastrella rosea*	Starfish & Brittlestars (excl Sunstar)	nd	133
CEND 086	2019	71	Goosefoot starfish	*Anseropoda Placenta*	Starfish & Brittlestars (excl Sunstar)	8	51
CEND 087	2019	71	Portunid crabs	Portunidae (indet.)	Crustacean (Anomura & Brachyura)	24	nd
CEND 088	2019	71	Brittlestar	*Ophiura ophiura*	Starfish & Brittlestars (excl Sunstar)	8	nd
CEND 089	2019	71	Shrimp	*Natantia* sp.	Crustacean (Natania & Stomatopoda)	12	nd
CEND 090	2019	71	Hermit Crab	*Pagurus* sp.	Crustacean (Anomura & Brachyura)	93	17
CEND 091	2019	71	Rigid cushion starfish	*Hippasteria phrygiana*	Starfish & Brittlestars (excl Sunstar)	11	nd
CEND 092	2019	43	Common Starfish	*Asterias rubens*	Starfish & Brittlestars (excl Sunstar)	12	nd
CEND 093	2019	43	Sandstar	*Astropecten irregularis*	Starfish & Brittlestars (excl Sunstar)	14	nd
CEND 094	2019	43	Rigid cushion starfish	*Hippasteria phrygiana*	Starfish & Brittlestars (excl Sunstar)	nd	nd
CEND 095	2019	43	Hermit Crab	*Pagurus bernhardus*	Crustacean (Anomura & Brachyura)	11	8
CEND 096	2019	43	Flying crab	*Liocarcinus holsatus*	Crustacean (Anomura & Brachyura)	nd	nd
CEND 097	2019	43	Anemone	Actiniaria (indet.)	Actiniaria	nd	nd
CEND 098	2019	43	Sea urchin	*Echinus* sp.	Sea urchin	26	nd
CEND 099	2019	43	Whelk	Buccinidae (indet.)	Molluscs	nd	nd
CEND 100	2019	43	Dead-man’s fingers	*Alyconium digitatum*	Sessile colonial fauna	23	nd
CEND 101	2019	43	Sea urchin	*Echinus acutus*	Sea urchin	9	nd
CEND 102	2019	43	Slender colus	*Colus gracilus*	Molluscs	18	48
CEND 103	2019	43	Whelk	Buccinidae (indet.)	Molluscs	20	15
CEND 104	2019	43	Whelk	Buccinidae (indet.)	Molluscs	nd	38
CEND 105	2019	43	Sea mouse	*Aphrodita aculeata*	Polychaeta	30	Nd
CEND 106	2019	13	Rigid cushion starfish	*Hippasteria phrygiana*	Starfish & Brittlestars (excl Sunstar)	nd	Nd
CEND 107	2019	13	Hermit Crab	*Pagurus* sp.	Crustacean (Anomura & Brachyura)	nd	Nd
CEND 108	2019	13	Sea mouse	*Aphrodita aculeata*	Polychaeta	587	357
CEND 109	2019	13	Dead-man’s fingers	*Alyconium digitatum*	Sessile colonial fauna	nd	nd
CEND 110	2019	13	Brittlestar	*Ophiura ophiura*	Starfish & Brittlestars (excl Sunstar)	8	nd
CEND 111	2019	13	Anemone	Actiniaria (indet.)	Actiniaria	31	11
CEND 112	2019	13	Common Starfish	*Asterias rubens*	Starfish & Brittlestars (excl Sunstar)	34	10
CEND 113	2019	13	Plumose anemone	*Metridium senile*	Actiniaria	9	27
CEND 114	2019	13	Bivalve	Bivalvia (indet.)	Molluscs	28	17
CEND 115	2019	1	Dead-man’s fingers	*Alcyonium digitatum*	Sessile colonial fauna	nd	nd
CEND 116	2019	1	Brittlestar	*Ophiura ophiura*	Starfish & Brittlestars (excl Sunstar)	8	nd
CEND 117	2019	1	Sea mouse	*Aphrodita aculeata*	Polychaeta	nd	nd
CEND 118	2019	1	Hermit crab	*Pagurus bernhardus*	Crustacean (Anomura & Brachyura)	nd	nd
CEND 119	2019	1	Common Starfish	*Asterias rubens*	Starfish & Brittlestars (excl Sunstar)	14	nd
CEND 120	2019	1	Green sea urchin	*Psammechinus miliaris*	Sea urchin	nd	nd
CEND 121	2019	1	Echiuran worm	*Echiura* sp.	Other	nd	nd
CEND 122	2019	1	Inachidae crab	*Inachus* sp.	Crustacean (Anomura & Brachyura)	66	13
CEND 123	2019	1	Anemone	Actiniaria (indet.)	Actiniaria	92	13
CEND 124	2019	1	Whelk	*Buccinum undatum*	Molluscs	1	nd
CEND 125	2019	1	Shrimp	*Crangon* sp. & *Rissoides desmarseti*	Crustacean (Natania & Stomatopoda)	nd	17
CEND 126	2019	1	Crabs	*Portunidae* sp. *& Ebalia* sp.	Crustacean (Anomura & Brachyura)	25	nd
CEND 127	2019	3	Hermit Crab	*Pagurus bernhardus*	Crustacean (Anomura & Brachyura)	nd	12
CEND 128	2019	3	Swimming crabs	*Liocarcinus holsatus & Liocarcinus depurator*	Crustacean (Anomura & Brachyura)	nd	9
CEND 129	2019	3	Common shore crab	*Carcinus maenas*	Crustacean (Anomura & Brachyura)	nd	nd
CEND 130	2019	3	Sea mouse	*Aphrodita aculeata*	Polychaeta	44	nd
CEND 131	2019	3	Sea chervil	*Alcyonidium diaphanum*	Sessile colonial fauna	1486	2091
CEND 132	2019	3	Common Starfish	*Asterias rubens*	Starfish & Brittlestars (excl Sunstar)	21	nd
CEND 133	2019	3	Brittlestar	*Ophiura ophiura*	Starfish & Brittlestars (excl Sunstar)	14	29
CEND 134	2019	3	Shrimp	*Crangon* sp.	Crustacean (Natania & Stomatopoda)	16	18
CEND 135	2019	3	Spider crab	*Macropodia* sp.	Crustacean (Anomura & Brachyura)	35	31
CEND 136	2019	3	Green sea urchin	*Psammechinus miliaris*	Sea urchin	10	19
CEND 137	2019	2	Common Starfish	*Asterias rubens*	Starfish & Brittlestars (excl Sunstar)	317	354
CEND 138	2019	2	Green sea urchin	*Psammechinus miliaris*	Sea urchin	377	257
CEND 139	2019	2	Sea chervil	*Alcyonidium diaphanum*	Sessile colonial fauna	2744	1461
CEND 140	2019	2	Brittlestar	*Ophiura ophiura*	Starfish & Brittlestars (excl Sunstar)	593	489
CEND 141	2019	2	Sea mouse	*Aphrodita aculeata*	Polychaeta	443	386
CEND 142	2019	2	Swimming crabs	*Liocarcinus* sp.	Crustacean (Anomura & Brachyura)	76	88
CEND 143	2019	2	Whelk	*Baccinum undatum*	Molluscs	133	110
CEND 144	2019	2	Sea chervil	*Alcyonidium diaphanum*	Sessile colonial fauna	562	288
CEND 145	2019	?	Rigid cushion starfish	*Hippasteria phrygiana*	Starfish & Brittlestars (excl Sunstar)	nd	nd
CEND 146	2019	7	Sea mouse	*Aphrodita aculeata*	Polychaeta	nd	nd
CEND 147	2019	7	Common Starfish	*Asterias rubens*	Starfish & Brittlestars (excl Sunstar)	nd	10
CEND 148	2019	7	Bloody Henry starfish	*Henricia oculata*	Starfish & Brittlestars (excl Sunstar)	nd	11
CEND 149	2019	7	Sunstar	*Crossaster papposus*	Sunstar	654	1275
CEND 156	2019	7	Swimming crabs	*Liocarcinus holsatus & Liocarcinus depurator*	Crustacean (Anomura & Brachyura)	nd	nd
CEND 157	2019	7	Masked crab	*Corystes cassivelaunus*	Crustacean (Anomura & Brachyura)	nd	nd
CEND 158	2019	7	Dead-man’s fingers	*Alcyonium digitatum*	Sessile colonial fauna	nd	nd
CEND 159	2019	7	Mermaids glove	*Haliclona oculata*	Sessile colonial fauna	21	nd
CEND 160	2019	4	Common starfish	*Asterias rubens*	Starfish & Brittlestars (excl Sunstar)	nd	nd
CEND 161	2019	4	Sea mouse	*Aphrodita aculeata*	Polychaeta	nd	nd
CEND 162	2019	4	Green sea urchin	*Psammechinus miliaris*	Sea urchin	nd	nd
CEND 163	2019	4	Hermit Crab	*Pagurus bernhardus*	Crustacean (Anomura & Brachyura)	nd	nd
CEND 164	2019	4	Brittlestar	*Ophiura ophiura*	Starfish & Brittlestars (excl Sunstar)	nd	nd
CEND 165	2019	4	Swimming crabs	*Liocarcinus holsatus & Liocarcinus depurator*	Crustacean (Anomura & Brachyura)	nd	15
CEND 166	2019	4	Circular Crab	*Atelecyclus* sp.	Crustacean (Anomura & Brachyura)	nd	nd
CEND 167	2019	4	Whelk	*Buccinum undatum*	Molluscs	nd	nd
CEND 168	2019	22	Rigid cushion starfish	*Hippasteria phrygiana*	Starfish & Brittlestars (excl Sunstar)	nd	nd
CEND 169	2019	22	Anemone	Actiniaria (indet.)	Actiniaria	14	45
CEND 170	2019	22	Sea mouse	*Aphrodita aculeata*	Polychaeta	37	46
CEND 171	2019	22	Sandstar	*Astropecten irregularis*	Starfish & Brittlestars (excl Sunstar)	nd	183
CEND 172	2019	22	Woody canoe bubble	*Scaphander lignarius*	Molluscs	nd	nd
CEND 173	2019	22	Seven-armed Starfish	*Luidia ciliaris*	Starfish & Brittlestars (excl Sunstar)	nd	nd
CEND 174	2019	22	Brittlestar	*Ophiura ophiura*	Starfish & Brittlestars (excl Sunstar)	nd	27
CEND 175	2019	22	Shrimp	Natantia (indet.)	Crustacean (Natania & Stomatopoda)	7	nd
CEND 176	2019	77	Sea mouse	*Aphrodita aculeata*	Polychaeta	12	13
CEND 177	2019	77	Rigid cushion starfish	*Hippasteria phrygiana*	Starfish & Brittlestars (excl Sunstar)	nd	nd
CEND 178	2019	77	Brittlestar	*Ophiura ophiura*	Starfish & Brittlestars (excl Sunstar)	nd	nd
CEND 179	2019	77	Sandstar	*Astropecten irregularis*	Starfish & Brittlestars (excl Sunstar)	nd	6
CEND 180	2019	77	Anemone	Actiniaria (indet.)	Actiniaria	22	72
CEND 181	2019	73	Sunstar	*Crossaster papposus*	Sunstar	1600	1102

**Table A2:** Table describing additional station information.

Station	Coordinates	Bottom Salinity	Surface Salinity	Bottom Temp. (°C)	Surface Temp. (°C)	Depth (m)	Date
1	51:42N 01:45E	33.7	34.5	19.6	19.8	33	11 August 2018
3	51:50N 03:37E	32.4	32.5	20.9	21	24	11 August 2018
4	52:49N 02:45E	34.4	34.4	17.6	17.6	38	12 August 2018
7	53:59N 00:15E	34.5	34.5	12.6	12.4	53	14 August 2018
13	54:56N 00:16W	34.7	34.5	7.4	16.3	77	14 August 2018
77	55:56N 01:27W	34.8	34.8	9.5	13.9	84	7 September 2018
22	55:35N 00:49W	34.9	34.9	7.4	15.3	96	7 September 2018
8	53:56N 01:17E	34.8	34.8	14.2	16.4	42	14 August 2018
1	51:45N 01:44E	35.1	35.0	18.8	19	30	7 August 2019
2	51:35N 02:46E	34.8	34.8	19.6	19.6	32	7 August 2019
3	51:50N 03:38E	33.5	33.4	20.1	20.2	27	8 August 2019
4	52:50N 02:46E	34.7	34.7	17.8	18.1	41	8 August 2019
7	53:60N 00:16E	34.4	34.4	13.1	14.5	55	11 August 2019
13	54:33N 00:02W	34.6	34.6	9.6	15.9	65	11 August 2019
22	55:35N 00:48W	34.9	34.5	8.4	16.2	99	18 August 2019
71	61:01N 00:60W	35.4	35.3	9.6	13.3	132	24 August 2019
43	57:19N 02:27E	35.1	34.8	7.7	16.9	84	28 August 2019
73	61:16N 00:34E	35.4	35.4	9.3	13.4	170	24 August 2019
50	57:43N 05:10E	35.3	34.0	8	16	110	27 August 2019
77	55:58N 01:25W	34.7	34.5	9.3	15	90	18 August 2019
